# Fast-setting Bone Cement in Total Knee Arthroplasty: A Case Series Looking at Safety and Short-term Radiological Outcomes

**DOI:** 10.5704/MOJ.2511.008

**Published:** 2025-11

**Authors:** P D’sa, S Mercer, S Ghosh, BK Thomas, L Atkinson, S Bajada, R Williams

**Affiliations:** Department of Trauma and Orthopaedics, Glangwili General Hospital (GGH), Carmarthen, United Kingdom

**Keywords:** total knee arthroplasty, safety, fast-setting bone cement, PALACOS® fast R+G, KSRESS score

## Abstract

**Introduction:**

Fast-setting high viscosity cement was introduced in the last decade, offering arthroplasty surgeons the benefit of shortened setting time. This could reduce the operating time, which may reduce the risk of infection and improve theatre efficiency. PALACOS® Fast R+G high viscosity cement has an average setting time of less than 6 minutes (30% faster than regular PALACOS® R+G) due to the lack of a waiting phase. The aim of this study was to investigate the safety of total knee arthroplasty performed using this fast-setting, high viscosity cement and short-term radiological outcomes.

**Material and Methods:**

This single surgeon case series looked at 344 primary TKAs performed using PALACOS® fast R+G cement from January 2016 to March 2020. Data were collected on patient demographics, perioperative events, and complications. Radiographs taken immediately post-operatively and at the one-year follow-up were analysed using the Knee Society Roentgenographic Evaluation and Scoring System (KSRESS).

**Results:**

This case series included 313 consecutive patients (31 bilateral) with a mean age of 70 years (range 44-93). A total of 237 patients (76%, 262 TKA patients) had a minimum one-year follow-up. No adverse events were noted perioperatively; ten patients had superficial wound issues and were managed successfully with wound care and/or oral antibiotics. Six (1.7%) patients underwent re-operation in the study period. These included one DAIR, one staged revision for deep infection, two revisions for instability, one manipulation under anaesthetic for stiffness, and one patella internal fixation for fracture. The mean combined valgus angle for the prosthesis was 183.1° (range 177.7° to 187.8°), indicating adequate alignment. At one-year follow-up, no radiographs demonstrated any new loosening or worsening of any previously noted radiolucent lines.

**Conclusion:**

This study reports the largest case series that looks at the use of fast-setting bone cement in primary TKA. It demonstrates good safety, as evidenced by a low re-operation rate, deep infection rate, and no adverse events during implantation. Fast-setting cement offers the promise of improving theatre efficiency and decreasing total running costs. Further studies are needed to provide data on improved theatre efficiency, cost savings and the longevity of implanted knees utilising this cement.

## Introduction

Cement remains the most commonly used method of implant fixation in total knee arthroplasty (TKA), with good long-term survival rates^1,2^. Fast-setting high viscosity cement was introduced in the last decade, offering arthroplasty surgeons the benefit of shortened setting times. These cements have altered handling characteristics compared to standard-setting high viscosity polymethylmethacrylate (PMMA) cements, thus resulting in a shortened mixing phase, moderate working time, and a very short hardening phase. This could reduce the operating time, which may reduce the risk of infection and improve theatre efficiency. PALACOS® Fast R+G high viscosity cement has an average setting time of less than 6 minutes (30% faster than regular PALACOS® R+G) due to the lack of a waiting phase^3^. It is licenced for use in primary joint arthroplasties as well as the second phase of two stage revision arthroplasty.

This study reports the safety and short-term radiological outcomes from a single surgeon, single implant series of fast-setting, antibiotic-loaded, high viscosity cement in primary TKA.

## Materials and Methods

Study design and eligibility criteria: All adult patients who underwent primary TKA using fast-setting bone cement [PALACOS® fast R+G, Heraeus Medical GmbH, Hanau, Germany] for any indications in the study period between January 2016 and March 2020 were included in the case series. Patients who underwent complex primary TKA or revision TKA were excluded. All procedures were performed by a fellowship-trained arthroplasty surgeon and senior author (RW) at a district general hospital. The study was approved by the local health board as a service evaluation study. Primary outcomes for safety analysis included intra-operative events. Secondary outcomes included death due to post-operative complications, return to the operating room, wound infections, and surgical complications, including re-operation/revision for any cause. Patients were identified using the theatre coding system. Data was collected from digital hospital records, including patient demographics and clinical outcomes, up to the start of data collection in May 2021.

Surgical technique: In accordance with local health board guidelines, all patients received intravenous antibiotics and 1 g of intravenous tranexamic acid at the induction of anaesthesia. Procedures were performed using a pneumatic tourniquet, which remained in use throughout. All patients underwent the standard measured resection technique with soft tissue balancing cemented arthroplasty. The medial parapatellar approach with or without patella resurfacing and the P.F.C.® SIGMA® Total Knee System [DePuy Synthes, Johnson and Johnson, Raynham, Massachusetts, U.S], either cruciate-retaining or posterior stabilized implants, were used. Bone resections were performed using an extramedullary tibial jig and an intramedullary femoral jig.

Cementing technique: All bony cuts were thoroughly washed with pulsatile lavage to clear all marrow and debris, followed by drying of the bone. Multiple drill holes were made into the tibial plateau and into any sclerotic bone surfaces on the distal femur to depths of 3 – 5mm using a 2.7mm diameter drill bit. Single mix PALACOS® fast R+G cement was used in all cases, which included nonvacuum mixing for 30 seconds as per the manufacturer’s instructions^3^. A cement gun was used for cement application to both bony cuts as well as the prosthesis undersurface, including keel and short stems. Both the tibia and femoral components were fixed in a single setting, and the knee was fully extended to apply pressure during cement curing. If the patella was resurfaced, it was cemented at the same sitting after the knee was extended and held with a patella clamp during cement curing.

Post-operative course: Thromboembolic prophylaxis included perioperative foot pumps and post-operative low molecular weight heparin (LMWH). Patients were discharged on either LMWH, or aspirin for four weeks based on their venous thromboembolism risk assessment score, as per local health board guidelines. All patients were managed using the enhanced recovery protocol after surgery as well as standard physiotherapy protocols. Most patients were discharged between days two and four post-operatively once they were deemed safe by the physiotherapy and surgical team. Patients were planned for review at the following periods post-operatively: six weeks, six months, one year, and five years. These reviews were conducted in a specialist arthroplasty practitioner-led clinic supervised by the operating surgeon. Patients underwent weight-bearing anteroposterior and lateral radiographs of the knee in the immediate post-operative period prior to discharge at the one-year and five-year follow-ups.

Radiographic assessment: Immediate post-operative weight-bearing radiographs were assessed using the Knee Society Roentgenographic Evaluation and Scoring System (KSRESS), a validated tool for evaluating TKA outcomes^4^. This method takes into account prosthesis alignment as well as prosthesis-to-bone fixation in both anterior-posterior and lateral views, which are considered to be the most important predictors of a successful TKA. The picture archiving and communication system (PACS) [InSight 8.0, Insignia’s Viewer for DICOM images, Insignia Medical Systems Limited, Hampshire, United Kingdom] viewer was used to complete the above analysis. All radiographic assessments and data collection were performed by three authors (PD, SG, BK) independent of the operating surgeon’s involvement. These assessments were performed independently and blindly. On the AP radiograph, a femoral component angle (angle alpha-α) and a tibial component angle (angle beta-β) were measured, and the two were then combined (α + β) to provide a total valgus angle for the prosthesis. On the lateral radiograph, the femoral flexion angle (angle gamma-γ; negative if the implant is in extension) and tibia slope angle (angle sigma-σ) were measured. The loosening score was noted in both views for each zone, and the total was added to obtain the final loosening score as per KSRESS. Weight-bearing radiographs taken at the one-year follow-up were assessed for any loosening or progression of radiolucent lines in any zones. All collected data was tabulated in Microsoft Excel [Microsoft Corporation, Redmond, Washington, US] and analysed using simple descriptive statistics.

Statistical analysis: All collected data were tabulated in Microsoft Excel [Microsoft Corporation, Redmond, Washington, US] and analysed using simple descriptive statistics. Data were analysed using Statistical Product and Service Solutions package 20.0 [IBM SPSS, Armonk, New York, USA]. For all statistical analyses, p values of <0.05 were considered significant. The intraclass correlation coefficient (ICC) statistic was used to analyse the interobserver and intra-observer agreement. ICC values were interpreted as follows: less than 0.40—poor, 0.40 to 0.59— fair, 0.60 to 0.74—good, and 0.75 to 1.00—excellent.

## Results

The case series included 313 consecutive patients with a mean age of 70 years. Patient demographics are tabulated below (see Table I). All patients had a six-week follow-up after surgery. A total of 237 patients (76%, 262 TKA patients) had a minimum one-year follow-up. There was no intra-operative anaphylaxis or death due to post-operative complications noted in the perioperative period for any case. There were no cases that needed return to the operating theatre for any reason or readmissions relating to complications from the procedure within the first 90 days of the procedure. Ten patients had superficial wound issues, such as a stitch abscess or minor wound gaping, which were managed successfully with wound care and/or oral antibiotics. Six (1.7%) patients underwent re-operation in the study period. The reasons for re-operation are listed below (Table II). Two patients died of unrelated causes in the study period, more than two years after their surgery.

**Table I:** Patient Demographics (N=313 patients, 344 knees).

Demographic		N (Percentage)
Sex	Male	151 (48%)
	Female	162 (52%)
Age in years, Mean (Range)	70 years (44 – 93)	
Laterality	Unilateral	282 knees (82%)
	Bilateral same sitting	8 (16 knees, 5%)
	Bilateral different sitting	23 (46 knees, 13%)
Implant specifics	Patella resurfaced	23 knees (7%)
	Patella not resurfaced	321 knees (93%)
	Cruciate retaining	309 knees (90%)
	Posterior stabilised	35 knees (10%)

**Table II:** Number, type, and reason for reoperation and current clinical status of patients from the case series.

Type of re-operation	Reason of re-operation	Current clinical status
1-DAIR	Acute hematogenous infection after 2 years post-surgery	Symptom-free at 1.5 years
1-Two-stage revision	Persistent deep infection after failed DAIR at 5 months post-surgery	Symptom-free at 5 years
2-Single-stage revision	Symptomatic instability at 1-year	Symptom-free at 0.5 and 2 years
1-MUA	Stiffness at 3 months post-op	Improved range of motion, 3 years post MUA
1-Patella internal fixation	Traumatic Fracture of patella, 1-year post-op	Fracture united, 2 years post-op symptom-free

Abbreviations - DAIR: debridement, antibiotics, irrigation and retention of implants, MUA: manipulation under anaesthetic

**Fig. 1:**
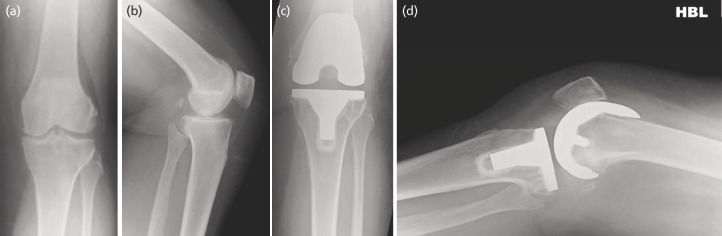
Patient 1, (a) pre-operative antero-posterior radiograph, (b) pre-operative lateral radiograph, (c) post-operative antero-posterior radiograph at one-year follow-up; showing stable isolated tibial lucency in zone 4, and (d) post-operative lateral radiograph at one-year follow-up.

**Fig. 2:**
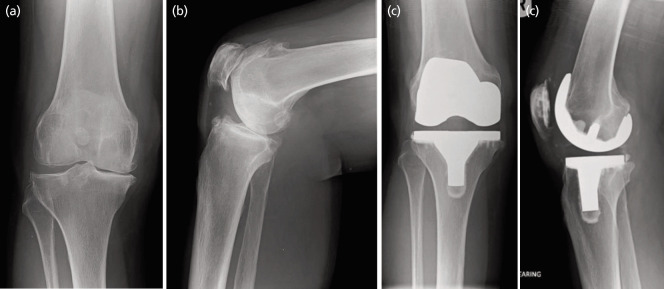
Patient 2, (a) pre-operative antero-posterior radiograph, (b) pre-operative lateral radiograph, (c) post-operative antero-posterior radiograph at one-year follow-up, and (d) post-operative lateral radiograph at one-year follow-up.

In the immediate post-operative radiographs, no TKAs demonstrated radiolucent lines in any femoral zones. Four TKAs were noted to have isolated tibial lucencies (three knees in zone 1 of 1mm in height and 3mm wide = score 3 each; one knee in zone 4 of 1mm height and 3mm wide = score 3). The mean and range for the radiographic angles are tabulated below (Table III). The mean combined valgus angle for the prosthesis was 183.1° (range 177.7° to 187.8°), indicating adequate alignment. The intraclass correlation coefficient value for interobserver agreement was excellent for all angles (mean=0.893, range=0.753 – 0.956), except the beta angle, for which it was good at 0.72. The ICC values for intra-observer agreement were excellent for all angles (mean=0.911, range= 0.841 – .953). At the one-year follow-up, no TKAs demonstrated new loosening or progression of previously noted radiolucent lines. Pre-operative and follow up antero-posterior and lateral radiographs of two patients from our series (Fig. 1, of patient 1 with stable isolated tibial lucency in zone 4; Fig. 2, of patient 2).

**Table III:** Radiographic assessment of immediate post-operative weight bearing radiographs as per KSRESS (knee society roentgenographic evaluation and scoring system).

Angle	Mean Value	Range
α	95.3°	92.7° to 99.7°
β	87.9°	83.9° to 89.8°
α + β (combined valgus)	183.1°	177.7° to 187.8°
γ *	2.9°	-6.5° to 8.3°
σ	85.5°	78° to 89.6°

Note - *(γ: angle gamma - negative if femoral component is in extension in sagittal alignment)

## Discussion

This study reports that using fast-setting high-viscosity bone cement in primary TKA demonstrates good safety. There were no cases of intra-operative anaphylaxis or death due to post-operative complications. Deep infection rates, re-operation rates and overall complications in the short term were low.

Fast-setting high-viscosity PMMA cement has gained popularity in arthroplasty due to its improved handling properties, reduced setting times, improved theatre efficiency, and overall cost savings. It is well established that longer operative times correlate with an increased risk of prosthetic infection in joint replacement surgery^5,6^. In this study, only two cases of deep infection (0.6%) were recorded and successfully treated.

Caraan *et al*, in 2017, studied the two fast setting PMMA bone cements available, which included PALACOS® fast R+G and CMW 2G, and compared their characteristics with the standard-setting setting cement PALACOS® R+G, which is currently considered the gold standard^7^. Their experimental study concluded that both fast-setting cements are suitable for arthroplasty without compromising mechanical integrity and are compliant with international standards. PALACOS® fast R+G alters the handling properties of the cement by increasing the powder to liquid ratio (2.550:1) in combination with an increased benzoyl peroxide content^3^. PALACOS® fast R+G was workable immediately after the mixing phase of 30 seconds, and there was no waiting phase. This is said to remain in the working phase until four minutes from mixing and sets at roughly five to six minutes. However, published data on its safety in knee arthroplasty remain limited.

Gallart *et al* looked at the use of fast setting bone cement in total hip arthroplasty using a Cemex system fast Tecres®^8,9^. This was a small, randomised double-blinded trial with 20 patients per group comparing standard versus fast cement. On average, they saved two minutes of surgical time per component implantation. Their two-year follow-up showed no difference in the radiographic quality of the cement mantle between groups. Compared to this, however, PALACOS® fast R+G boasts further time savings, as it sets at approximately six minutes compared to the standard PALACOS® R+G average setting time of 12 minutes. This could save six minutes per case in single-stage implantation and up to 12 minutes in cemented hip arthroplasty or sequential TKA. This amounts to a potential minimum cost savings of 200-250 USD per patient in operating room costs (36USD per minute)^10^. However, due to the retrospective nature of our study, we were unable to collect data on actual operative time savings. This would be an area for further investigation.

Howie *et al*^11^ recently published a comparative retrospective cohort study of 160 patients, evaluating blood loss, operative time, and cost savings. This was a single-centre study where they categorized five cements used in two categories, slow and fast setting. Their results found that the average blood loss was greater in the fast-setting bone cement group (160.0 vs 126.4mL; p=0.0009), and there was no difference in operative times between the groups (88.2 vs 89.2 min; p=0.99); however, fewer bags of cement were used for the fast cohort (1.3 vs 1.8 bags; p<0.0001). Their results also found that the fast group was significantly cheaper on average per patient when comparing between antibiotic bone cements (p=0.007). They concluded that decreased usage of fast cement did not result in any different post-operative outcomes compared to slow cements. In comparison to this study, our larger single-implant, single-surgeon case series only looks at the results from antibiotic-loaded fast cement but lacks the comparator group and data on actual time savings. These will need to be examined in more detail in large prospective comparative studies.

The use of fast-setting cement has been mentioned to be increasing in the literature as well as UK/Australian joint registries^1,2,7^. However, no data are available to exactly quantify its current usage in various arthroplasty procedures. The manufacturer recommends non-vacuum mixing due to the short 30-second mixing time and absence of a waiting phase. It is recommended for use in primary joint arthroplasty and the second stage of a two-stage revision for total joint arthroplasty, but not for use in femoral stem implantation due to the risk of incomplete insertion and early setting of cement.

The implant used in this cohort – the P.F.C.® SIGMA® Total Knee System [Sigma 23R, cemented, CoCr tibial tray, curved standard polyethylene insert, oval patella, DePuy Synthes, Johnson and Johnson, Raynham, Massachusetts, U.S] is a 10A* ODEP (Orthopaedic Data Evaluation Panel, UK)-rated implant and has had good long-term results, as evidenced by national joint registry (NJR) data^12^. On UK NJR, Kaplan‒Meier estimates of cumulative revision are at one to three years of 0.4 to 1.5%^1^. In this series, six (1.7%) patients underwent re-operation in the study period, which included four revisions and two other procedures.

To the best of our knowledge, this is the largest case series reporting the use of fast-setting cement in TKA, and we have demonstrated excellent safety. The early re-operation rate was low, with only two cases of infection and two cases of instability. However, there are several limitations to this study, including the lack of functional outcome scores, retrospective design, and lack of medium-term/long-term follow-up data. Due to the lack of data on a comparative cohort of TKR performed using standard PALACOS® R+G cement, we are unable to present any results on the theatre efficiency and cost savings. The lack of follow-up is mainly secondary to the disruption of elective services due to the COVID-19 pandemic, and we are currently looking into arranging further follow-up of these patients and collecting medium-term data. Functional scores were not collected during the early period of the study; however, this has been made a routine practice; hence, they are not included in this study. The strengths of this study include a large sample size, a single implant, and a single surgeon series of over a four-year period, which allows for a homogenous surgical and rehabilitation technique between patients. In addition, the selection bias was reduced by ensuring a consecutive series of patients. A large prospective randomised controlled trial aiming to look at the cost-effectiveness, impact on theatre efficiency, functional outcomes, radiological outcomes, and impact on patient-reported outcome measures would be able to overcome the above limitations and reveal the true impact of this fast-setting bone cement use in total joint arthroplasty.

## Conclusion

This study reports good safety when using a fast-setting cement to perform primary TKA, with the promise of improving theatre efficiency and reducing the total running costs over the long term. Further medium to long-term radiological and functional outcome results will be needed to provide further data on the durability of these implanted knees using fast-setting cement.
